# Analyzing the role of reoperation in recurrent glioblastoma: a 15-year retrospective study in a single institution

**DOI:** 10.1186/s12957-022-02852-3

**Published:** 2022-12-04

**Authors:** Víctor González, Marta Brell, José Fuster, Lesmes Moratinos, Daniel Alegre, Sofía López, Javier Ibáñez

**Affiliations:** 1 Neurosurgical Department, Hospital Son Espases, Carretera de Valldemossa, 79, 07120 Palma, Illes Balears Spain; 2 Oncology Department, Hospital Son Espases, Carretera de Valldemossa, 79, 07120 Palma, Illes Balears Spain

**Keywords:** Glioblastoma, Recurrent, Reoperation, Survival

## Abstract

**Background:**

Multiple treatment options at glioblastoma progression exist, including reintervention, reirradiation, additional systemic therapy, and novel strategies. No alternative has been proven to be superior in terms of postprogression survival (PPS). A second surgery has shown conflicting evidence in the literature regarding its prognostic impact, possibly affected by selection bias, and might benefit a sparse subset of patients with recurrent glioblastoma. The present study aims to determine the prognostic influence of salvage procedures in a cohort of patients treated in the same institution over 15 years.

**Methods:**

Three hundred and fifty patients with confirmed primary glioblastoma diagnosed and treated between 2005 and 2019 were selected. To examine the role of reoperation, we intended to create comparable groups, previously excluding all diagnostic biopsies and patients who were not actively treated after the first surgery or at disease progression. Uni- and multivariate Cox proportional hazards regression models were employed, considering reintervention as a time-fixed or time-dependent covariate. The endpoints of the study were overall survival (OS) and PPS.

**Results:**

At progression, 33 patients received a second surgery and 84 were treated with chemotherapy only. Clinical variables were similar among groups. OS, but not PPS, was superior in the reintervention group. Treatment modality had no impact in our multivariate Cox regression models considering OS or PPS as the endpoint.

**Conclusions:**

The association of reoperation with improved prognosis in recurrent glioblastoma is unclear and may be influenced by selection bias. Regardless of our selective indications and high gross total resection rates in second procedures, we could not observe a survival advantage.

## Background

High-grade glioma remains one of the most frequent pathologies associated with poor prognosis, despite modest improvements in overall survival (OS) [1] and progression-free survival (PFS) after the introduction of the Stupp protocol [2] in recent years. This standard of care consists of maximal surgical resection whenever feasible, followed by radiotherapy and temozolomide (TMZ) concomitant and adjuvant schedules. Unfortunately, regardless of optimal treatment fulfillment, tumor regrowth is the rule in virtually all patients, with some options at this point that include different salvage chemotherapy schemes, reresection, additional radiotherapy, or tumor treating fields, alone or in combination [3–6].

As there is no established second-line chemotherapy regimen, patients are usually treated within investigational protocols. Bevacizumab is an agent commonly used in clinical practice for relapsed glioblastoma that has demonstrated high response rates [7, 8]. However, this short-lasting effect may be due to changes in vascular permeability. Nitrosurea-based therapeutic strategies (lomustine, fotemustine) have added limited survival effects, alone or in combination [9]. Newer strategies, such as immunotherapy, targeted agents, or novel radiation modalities, have yielded promising results in clinical trials, but further validation is needed [10].

A second surgery is a treatment option for a limited number of patients only due to poor clinical status or involvement of critical brain areas. The current data on the role of reoperation in recurrent glioblastoma are still lacking in high levels of clinical evidence, mainly owing to the retrospective nature of the majority of the studies, heterogeneity of the clinical scenario that always implies a strong selection bias, and lack of prospective data collection [11, 12]. This study aims to explore a prognostic role for reoperation in recurrent glioblastoma patients and to compare its benefits whether it is considered a standard, time-fixed covariate or a time-dependent covariate.

## Methods

### Patient selection

We retrospectively analyzed patients with new histopathological diagnoses of glioblastoma according to 2016 World Health Organization criteria [13] in our institution over a period between January 2005 and December 2019. The follow-up period ended on 12.31.2021. The study protocol was approved by the institutional ethics committee. Patients were divided based on the extent of the primary operation between resection and biopsy. Biopsies’ main indications were deep-seated or multicentric location, involvement of brain eloquent areas, and comorbidities that would preclude a debulking procedure. Our target study group included patients in whom surgical tumor resection was indicated, followed by ulterior complementary treatment; disease progression was detected and treated. Patients diagnosed only with biopsy, not treated within the Stupp protocol after the first surgery, or not actively treated after progression (best supportive care (BSC)) were excluded from the study group.

**Fig. 1:**
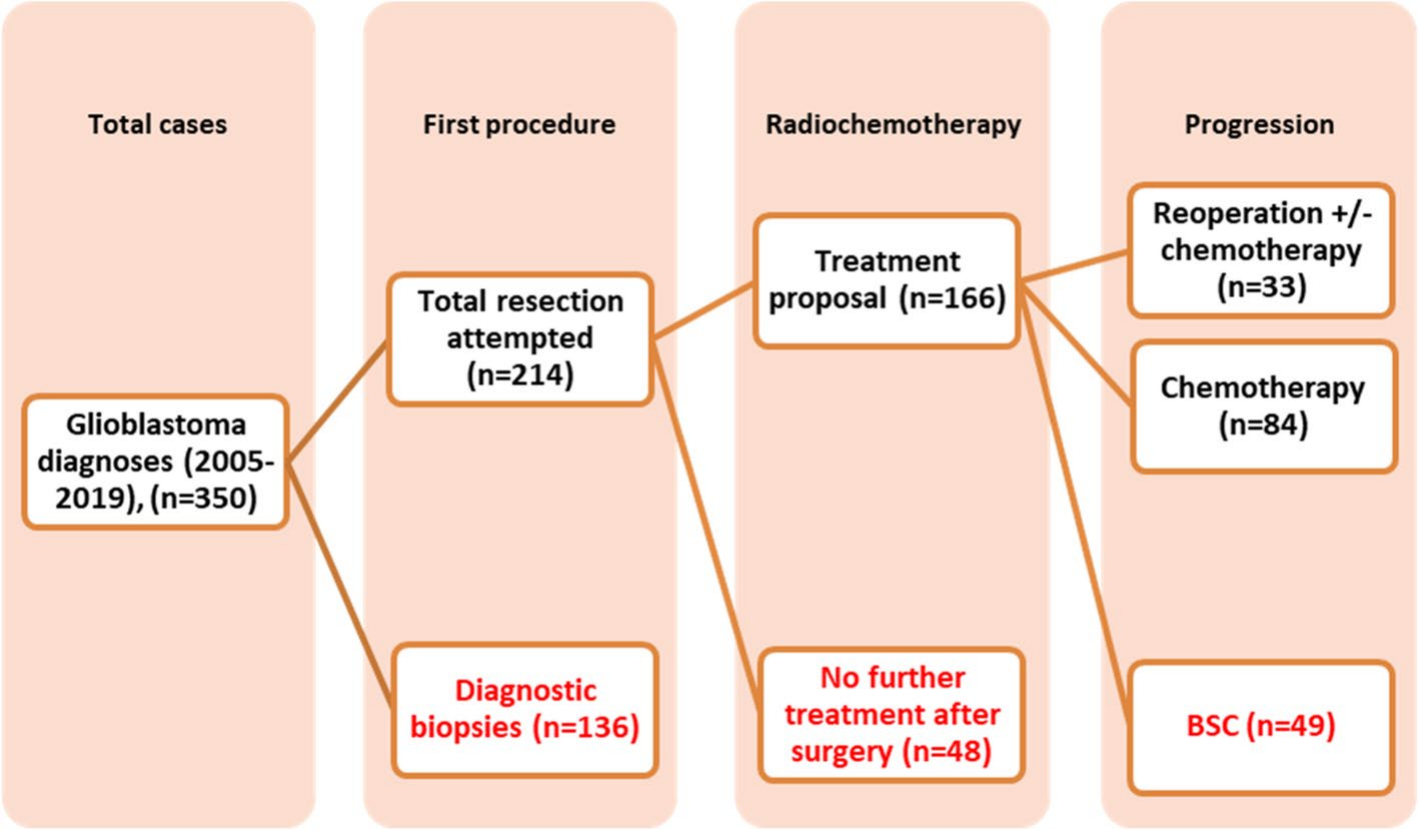
Flowchart diagram illustrating patient selection in our series

To determine the efficacy of a second surgery at glioblastoma progression, our group sample was stratified into two cohorts: (i) patients who underwent reresection generally followed by systemic treatment and (ii) patients treated with additional chemotherapy only. A flowchart diagram illustrates the patient selection process (Fig. 1).

All patients with recurrent disease were discussed in our multidisciplinary tumor board. Although a clear protocol in our institution is lacking, general considerations for reintervention include patients in good clinical condition with tumors that arise in the vicinity of the previous cavity and not involving eloquent cortical areas, basal ganglia, or diencephalic or brainstem structures, with a PFS generally superior to 9 months.

### Clinical variables

Medical records were evaluated in terms of age, sex, comorbidities, clinical symptoms, tumor location, and estimated volume in cubic centimeter based on the abc/_2_ method. The extent of resection (EOR) was categorized as total (GTR) or partial (STR) if any contrast-enhanced image remained in an early postoperative MRI scan. The use of surgical assistants was divided between conventional neuronavigation or “advanced” if 5-ALA fluorescence guidance or intraoperative MRI were employed. Patients’ clinical status was defined based on the Eastern Cooperative Oncology Group (ECOG) scale and was measured after the first procedure, at the first progression, after the second surgery, and at the second progression. Patients were divided into symptomatic (ECOG 2–3–4) and asymptomatic or mild symptoms (ECOG 0–1) groups. Completion of maximal first-line treatment was assessed. All recorded complications were grouped according to the Landriel-Ibañez classification [14]. Finally, we divided our population depending on the year in which they were diagnosed.

The date, clinical characteristics, and choice of treatment at progression were noted. Continuous variables such as age and PFS were also dichotomized, taking into account the median of our study group as the cutoff value.

### Outcomes

OS was defined as the time from initial diagnosis until death of any cause, considering censored patients who were still alive at the study endpoint or lost to follow-up. PFS was defined as the time from diagnosis to objective tumor recurrence in neuroimaging based on the RANO criteria [15]. MRIs with high suspicion of pseudoprogression [16] were monitored with follow-up images at 8–12 weeks. Postprogression survival (PPS), defined as the time from tumor recurrence to death of any cause, was also considered.

### Statistical analysis

The statistical software used for the analyses was SPSS 22.0 (IBM, Armonk, NY). Categorical variables are presented as frequencies and percentages, and continuous variables are presented as medians and ranges. *χ*^2^ and Mann–Whitney *U* tests were conducted for intergroup comparisons. Survival analysis was performed using the log-rank test and illustrated by Kaplan–Meier survival curves. Univariate and multivariate Cox proportional hazards regression models were used to adjust for confounders in survival, obtaining hazard ratios (HR) and 95% confidence intervals (CI) [17]. *p* values less than or equal to 0.05 were considered significant.

The effect of a second surgery on OS and PPS in our multivariate models was evaluated in a classical manner first, considering reoperation as a time-fixed covariate (i.e., repeat resection status was known at initial diagnosis) and as a time-dependent covariate afterward (all patients belonged to the nonoperative group at the initial study, and it was only when they received a second operation that they crossed over to the reoperation group) [18].

## Results

### Descriptive statistics and intergroup comparison

Among the 350 patients with de novo glioblastoma diagnosis, total resection was attempted in 214 cases (61.1%). We excluded 136 patients who were diagnosed by open or stereotactic biopsies. Due to different clinical reasons, 48 patients did not receive further oncological treatment in our institution after surgical resection and were also excluded from the survival analysis. All patients were treated with 60 Gy of radiotherapy and completed a variable number of temozolomide adjuvant cycles. At progression, a combination of a second procedure followed by different chemotherapy schemes was elected in 33 cases (group i), 84 patients received chemotherapy (group ii), and exclusion was performed in the remaining 49 cases receiving BSC.

The main characteristics of the 117 patients (group i + ii) included in the study sample are summarized in Table 1. The mean age of the cohort was 58 years. There were significant differences between groups in terms of EOR*,* completeness of the Stupp protocol, clinical status at disease relapse, type, and radiological pattern of progression. We could not detect differences in age, sex, tumor volume or location, medical comorbidities, or complications after the first surgery. No differences were identified in molecular markers (p53, EGFR amplification, Ki67, *IDH*, and *MGMT* promoter methylation status). These facts demonstrate the homogeneity of the selected cohort.

**Table 1:** Clinical characteristics of the compared groups (*n* = 117 patients)

Variable of interest	Reintervention (*n* = 33)	Chemotherapy (*n* = 84)	Statistical test	*p value*
Age (years)			MW-U	0.085
Mean	52.91	57.70		
Median	55.00	58.00		
Range	7–70	31–76		
Sex			*χ*^2^	0.364
Male	17 (51.5%)	51 (60.7%)		
Female	16 (48.5%)	33 (39.3%)		
Comorbidities			*χ*^2^	0.351
Present	7 (21.2%)	25 (29.8%)		
Absent	26 (78.8%)	59 (70.2%)		
Volume (cm^3^)			MW-U	0.501
Mean	30.61	33.52		
Median	22.90	24.10		
Range	1.0–111.9	1.6–116.5		
Resection at first surgery			*χ*^2^	**0.036**
GTR	29 (87.9%)			
STR	4 (12.1%)			
Surgical assistants			*χ*^2^	0.415
None	11 (33.3%)	15 (17.9%)		
Navigation	12 (36.4%)	30 (35.7%)		
Advanced	10 (30.3%)	39 (46.4%)		
IDH status			*χ*^2^	0.922
IDH-wildtype	16 (48.5%)	44 (52.4%)		
IDH-mutant	1 (3.0%)	2 (2.4%)		
Not studied	16 (48.5%)	38 (45.2%)		
Complications at first surgery			*χ*^2^	0.676
None	26 (78.8%)	61 (72.6%)		
Grade I	4 (12.1%)	15 (17.8%)		
Grade II	2 (6.1%)	4 (4.8%)		
Grade III	1 (3.0%)	4 (4.8%)		
Grade IV	0 (0.0%)	0 (0.0%)		
ECOG 1			*χ*^2^	0.295
Asymptomatic/mild	30 (90.9%)	70 (83.3%)		
Symptomatic	3 (9.1%)	14 (16.7%)		
Chemotherapy (TMZ)			*χ*^2^	**0.001**
Complete Stupp	28 (84.8%)	40 (47.6%)		
Incomplete Stupp	5 (15.2%)	44 (52.4%)		
Progression			*χ*^2^	**0.020**
Only radiological	24 (72.7%)	31 (36.9%)		
Clinical	9 (27.3%)	53 (63.1%)		
ECOG 2			*χ*^2^	**0.001**
Asymptomatic/mild	27 (81.8%)	45 (53.6%)		
Symptomatic	6 (18.2%)	39 (46.4%)		

*GTR* gross total resection, *STR* subtotal resection, *ECOG 1* ECOG after the first procedure, *ECOG 2* ECOG at progression, *MW-U* Mann–Whitney *U* test, *χ*^*2*^ chi-square test

**Fig. 2:**
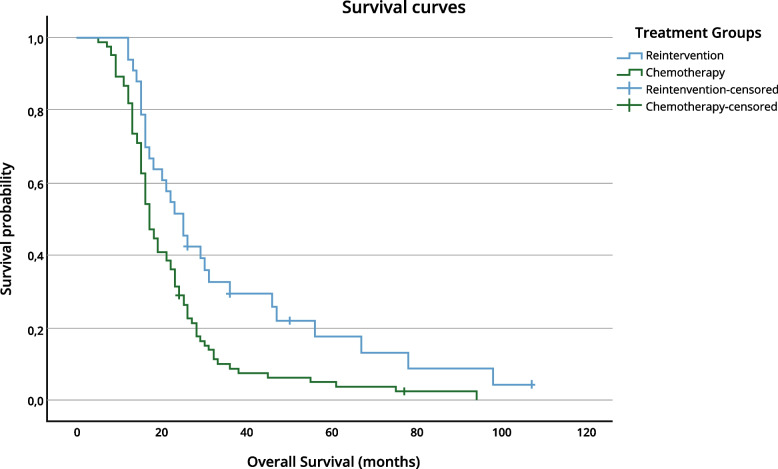
Kaplan–Meier survival curves comparing OS between treatment groups (log-rank test, *p* = 0.004)

**Fig. 3:**
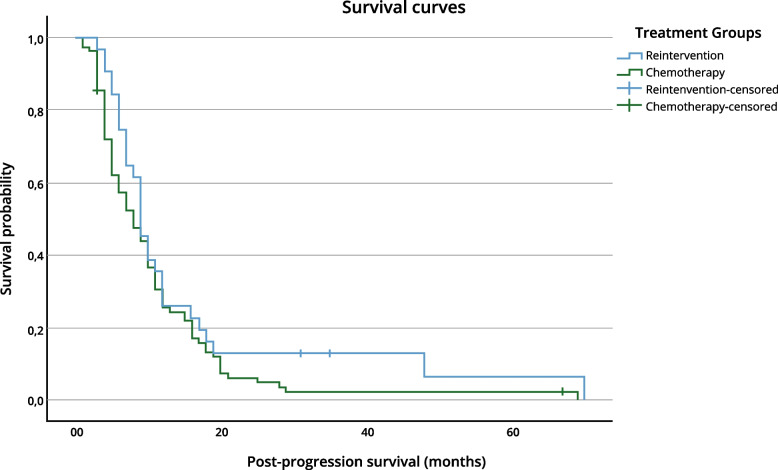
Kaplan–Meier survival curves comparing PPS between treatment groups (log-rank test, *p* = 0.143)

Among patients who underwent reintervention, the median OS was 25 months compared to 17 months in the nonsurgical group (log-rank test, *p* = 0.004). PFS differed significantly between cohorts (median 12 months vs. 9 months; *p* = 0.002). The median PPS was not significantly different between the surgical group (9 months) and the systemic treatment group (8 months) (*p* = 0.143). The Kaplan–Meier survival curves are illustrated in Figs. 2 and 3.

### Classic univariate and multivariate models

Univariate Cox proportional hazards analysis was performed to identify the significant influencing variables in prognosis. The results revealed that receiving at least six cycles of temozolomide (*p* < 0.001), having a PFS equal to or higher than the median of our group (10 months, *p* < 0.001), having a low score on the ECOG scale at progression (*p* = 0.002), and belonging to the reintervention group (*p* = 0.006) were significantly associated with OS. Considering PPS as the endpoint, our results reveal that patients in good clinical condition at progression (*p* = 0.002) and cases diagnosed and treated in recent years (2012–2019, *p* = 0.021) are associated with longer PPS. Modality of treatment (*p* = 0.166) or higher PFS (*p* = 0.836) were no longer associated with survival benefits. The complete results are summarized in Tables 2 and 3.

**Table 2:** Univariate Cox regression model for OS in 117 patients

Variable of interest	No. of events/no. of patients (*n* = 117)	HR (CI 95%)	*p* value
Age (years)			0.351
< 58	53/56	0.83 (0.57–1.03)	
≥ 58	58/61	1	
Comorbidities			0.595
Absent	80/85	0.89 (0.58–1.35)	
Present	31/32	1	
Volume (cm^3^)			0.270
< 24	56/58	0.80 (0.55–1.18)	
≥ 24	55/59	1	
Surgical assistants			0.961
Advanced	44/49	0.93 (0.57–1.52)	0.782
Navigation	41/42	0.94 (0.57–1.55)	0.961
None	26/26	1	
Complications			0.741
None	82/87	0.93 (0.60–1.42)	
Any	29/30	1	
Resection at first surgery			0.179
GTR	83/87	0.74 (0.47–1.14)	
STR	28/30	1	
ECOG 1			0.096
Asymptomatic/mild	94/100	0.64 (0.38–1.08)	
Symptomatic	17/17	1	
First surgery date			0.148
Recent (2012–2019)	77/83	0.74 (0.49–1.11)	
Old (2005–2011)	34/34	1	
Chemotherapy			** < 0.001**
Complete Stupp	63/68	0.48 (0.32–0.71)	
Incomplete Stupp	48/49	1	
ECOG 2			**0.002**
Asymptomatic/mild	67/72	0.54 (0.37–0.80)	
Symptomatic	44/45	1	** < 0.001**
PFS (months)			
≥ 10	54/59	0.39 (0.26–0.58)	
< 10	57/58	1	
Treatment groups			**0.006**
Reintervention	29/33	0.54 (0.34–0.83)	
Chemotherapy	82/84	1	

*GTR* gross total resection, *STR* subtotal resection, *ECOG 1* ECOG after the first procedure, *ECOG 2* ECOG at progression, *PFS* progression-free survival

**Table 3:** Univariate Cox regression model for PPS in 117 patients

Variable of interest	No. of events/no. of patients (*n* = 117)	HR (CI 95%)	*p* value
Age (years)			0.235
< 58	53/56	0.89 (0.73–1.07)	
≥ 58	58/61	1	
First surgery date			**0.021**
Recent (2012–2019)	77/83	0.61 (0.40–0.92)	
Old (2005–2011)	34/34	1	
Resection at first surgery			0.492
GTR	83/87	0.85 (0.55–1.32)	
STR	28/30	1	
Chemotherapy			0.791
Complete Stupp	63/68	0.95 (0.64–1.38)	
Incomplete Stupp	47/49	1	
ECOG 1			0.219
Asymptomatic/mild	94/100	0.71 (0.42–1.21)	
Symptomatic	17/17	1	
ECOG 2			**0.002**
Asymptomatic/mild	67/72	0.52 (0.35–0.78)	
Symptomatic	44/45	1	
PFS (months)			0.836
≥ 10	54/59	0.96 (0.65–1.40)	
< 10	57/58	1	
Treatment groups			0.166
Reintervention	29/33	0.73 (0.47–1.13)	
Chemotherapy	82/84	1	

*GTR* gross total resection, *STR* subtotal resection, *ECOG 1* ECOG after the first procedure, *ECOG 2* ECOG at progression, *PFS* progression-free survival

The multivariate Cox proportional hazards regression model revealed that no differences were encountered regarding OS in the modality of treatment at progression (HR 0.75; CI 95% 0.46–1.21; *p* = 0.248), adjusting for the most significant covariates. A longer progression-free interval (HR 0.38; CI 95% 0.20–0.74; *p* = 0.004) seems to be the only variable statistically associated with superior OS in our group (Table 4). There is no longer such an association if we perform the multivariable model taking PPS as the endpoint (HR 1.12; CI 95% 0.80–1.82; *p* = 0.356). In this setting, a good clinical status at progression or being operated on in the recent years of the study (2012–2019) appears to favor PPS (Table 5).

**Table 4:** Multivariate Cox regression model for OS in 117 patients

Variable of interest	No. of events/no. of patients (*n* = 117)	HR (CI 95%)	*p* value
Treatment groups			0.248
Reintervention	29/33	**0.75** (0.46–1.21)	
Chemotherapy	82/84	1	
Chemotherapy			0.709
Complete Stupp	63/68	0.88 (0.46–1.68)	
Incomplete Stupp	47/49	1	
ECOG 1			0.261
Asymptomatic/mild	94/100	0.72 (0.41–1.26)	
Symptomatic	17/17	1	
ECOG 2			0.061
Asymptomatic/mild	67/72	0.67 (0.44–1.01)	
Symptomatic	44/45	1	
PFS (months)			
≥ 10	54/59	0.38 (0.20–0.74)	**0.004**
< 10	57/58	1	

*ECOG 1* ECOG after the first procedure, *ECOG 2* ECOG at progression, *PFS* progression-free survival

**Table 5:** Multivariate Cox regression model for PPS in 117 patients

Variable of interest	No. of events/no. of patients (*n* = 117)	HR (CI 95%)	*p* value
Treatment groups			0.277
Reintervention	29/33	0.76 (0.47–1.24)	
Chemotherapy	82/84	1	
First surgery date			**0.018**
Recent (2012–2019)	77/83	0.59 (0.39–0.91)	
Old (2005–2011)	34/34	1	
ECOG 2			**0.006**
Asymptomatic/mild	67/72	0.55 (0.36–0.84)	
Symptomatic	44/45	1	
PFS (months)			
≥ 10	54/59	1.12 (0.80–1.82)	0.356
< 10	57/58	1	

*ECOG 2* ECOG at progression, *PFS* progression-free survival

### Multivariate model with reoperation as a time-dependent covariate

An analog multivariate analysis was performed, considering reintervention as a time-dependent covariate and controlling it for known predictors of OS. In this setting, we conclude that a longer progression-free interval (HR, 0.43; CI 95% 0.22–0.83; *p* = 0.012) and low scores (0 or 1) on the ECOG scale at progression (HR, 0.57; CI 95% 0.37–0.88; *p* = 0.011) continue to influence OS independently, but once we incorporate the timing of resection, it seems that a second surgery may negatively affect survival (HR, 1.76; CI 95% 1.10–2.80; *p* = 0.017). The complete results are summarized in Table 6.

**Table 6:** Multivariate Cox regression model for OS considering reoperation as a time-dependent covariate in 117 patients

Variable of interest	No. of events/no. of patients (*n* = 117)	HR (CI 95%)	*p* value
Reintervention as a time-dependent covariate			**0.017**
Reintervention	29/33	**1.76 (1.10–2.80)**	
No reintervention	82/84	1	
Chemotherapy			0.687
Complete Stupp	63/68	0.87 (0.46–1.65)	
Incomplete Stupp	47/49	1	
ECOG 1			0.185
Asymptomatic/mild	94/100	0.68 (0.39–1.19)	
Symptomatic	17/17	1	
ECOG 2			**0.011**
Asymptomatic/mild	67/72	0.57 (0.37–0.88)	
Symptomatic	44/45	1	
PFS (months)			**0.012**
≥ 10	54/59	0.43 (0.22–0.83)	
< 10	57/58	1	

*ECOG 1* ECOG after the first procedure, *ECOG 2* ECOG at progression, *PFS* progression-free survival

## Discussion

The treatment of recurrent glioblastoma remains a *challenging clinical decision*. Different attempts have been made to guide whether to proceed with surgical management at progression. Scoring systems based on patients’ clinical status, tumor volume, and involvement of eloquent cortex or ependymal tissue were developed [19–21]. Literature reviews identify factors associated with better prognosis and generally correlate second procedures with a survival advantage in selected candidates but lack the determination of concrete indications [22–24].

The median values for OS and PFS in our series are in accordance with recent literature reviews [21]. Our rate for repeated surgery (15.4%) is slightly inferior when compared to the percentages reported in other studies [22, 25–28]. This may be influenced by *different indications* for a “redo” procedure among centers. We do not usually consider a second surgery for patients who have previously received a partial resection. However, some reports include patients who have had an incomplete first resection or even biopsies in the “surgical group” [26, 29]. Our group also does not consider reintervention in tumors that spread in a distal, contralateral, or multicentric manner [30]. Multiple reresections are seldom considered, even though some authors found improvements in OS with repetitive surgeries [31]. Some series report a high incidence of patients suffering from new neurological deficits before the second operation [26]; however, our main indication (24 patients, 73%) was radiological tumor progression with minimal symptoms. Differences in indications among institutions may over- or underestimate the clinical usefulness of multiple procedures. Our *gross total resection rate* (29 cases, 87.8%) in the second surgeries is much higher than that in other reports [25, 32–35] and may be due to our limited surgical indications and intraoperative MRI or 5-aminolevulinic acid fluorescence guidance. Although completeness of resection in recurrent tumors has been identified as an independent factor associated with improved survival [27, 32, 33, 35–38], our study failed to appreciate this. Our complication rate after the second procedure (4 of 33 patients; 12%) was within acceptable percentages and in accordance with other reports [28, 33]. Despite adequate clinical results in reoperated patients, we failed to demonstrate improvements in survival. In fact, when reintervention was examined as a time-dependent covariate, it seemed to counteract survival (HR 1.76; *p* = 0.017).

Due to the heterogeneity of clinical practice, it is difficult to achieve *comparable groups*. We ultimately created two cohorts according to the treatment received, having previously excluded from the analysis all patients diagnosed by biopsy, not treated with the Stupp protocol or candidates for BSC at disease relapse, making both groups more homogeneous. Minimal differences in age and comorbidities were found between cohorts. In addition, it is not uncommon to include patients who received biopsy as the first treatment [29] or BSC at progression [21, 39] in the nonoperative group, probably favoring selection bias and overestimating survival data [27].

We believe that studies that *support reintervention* are likely to introduce a strong selection bias that favors the best candidates for redo procedures [26–28, 31, 39, 40]. In several reports, greater OS in surgical cohorts may be more influenced by favorable clinical characteristics than the procedure itself. To better assess this question, a subgroup analysis was created by Tully et al., excluding patients in the control group who were very unlikely to be considered for reoperation, suggesting a much less significant effect of second procedures on prognosis [40]. However, the initial difference in OS was 10.9 months between the groups.

Furthermore, strategies to deal with *temporal bias* are well established in the literature and have been addressed in other medical areas. The time from the initial diagnosis to recurrence or reoperation is not predictable and, hence, reoperation is a time-dependent variable in nature. Time-dependent methodology has been used to determine prognosis in breast [41] or colon cancer [42]. The vast majority of studies consider reoperation as a fixed covariate, but as explained by Beyersmann et al. [43], ignoring timing in a *time-dependent covariate*, such as reintervention, will lead to erroneous findings, overestimating the survival benefit [44]. A few novel studies [45, 46] and even meta-analyses [47] account for the timing of repeat resection, reversing the relationship between OS and multiple surgical procedures when using the proper time-dependent methodology. We detected the same phenomenon in our time-dependent multivariate Cox regression, but the clinical interpretation for these results is yet to be determined and should be done judiciously. Nevertheless, we believe that treating reoperation as a time-dependent covariate is a basis for future work assessing multiple interventions in glioblastoma cases. One presumption for these dismal results may be related to the fact that a new surgical procedure does not counteract tumor progression distant to the contrast-enhanced lesion (i.e., T2WI changes) and might cause delays in the initiation of further systemic treatment. Moreover, this can be aggravated by any potential postoperative complication.

However, reports that *do not give reintervention a survival advantage* exist [34, 48–52]. De Bonis et al. reported similar PPS rates and found PPS to be higher in patients treated with chemotherapy alone than in those treated with surgery alone [34]. Michaelsen et al. compared patients who received only chemotherapy at progression with those receiving both treatments with no additional effect on survival [53]**.** Moreover, some studies did not measure the *outcomes from the date of progression* [27, 28]. For this reason, we employed PPS to determine if adding a second surgery benefits survival from the date of recurrence; having detected that, although initial differences in OS were found, treatment modality had little impact on this endpoint. Our study failed to perceive any relationship between longer PFS and PPS. This may reveal that patients with longer PFS, who usually complete the Stupp regime and receive active treatment at progression, may have longer OS. The choice of therapy at progression seems to have less impact on prognosis in our group.

Patients elected for second procedures may have significantly *longer PFS*, which seems to be the more influential variable that favors OS in our group. Other colleagues further validated this finding [23, 27, 28, 48, 54] and may reflect the usual practice of not offering salvage procedures to early progressions. Multivariate Cox regression methodology was performed to reduce the confounding effects of subrogated variables. The correlation between PFS and OS was maintained, treating reresection as a stationary or time-dependent covariate. Although some studies have determined that reoperation improves survival regardless of other prognostic factors [27, 40], we could not identify that outcome. Only longer PFS and good clinical status at progression proved to be protective effects for OS in our multivariable model.

Although reresection has yielded disappointing results in our group, it may still have a role in some patients to allow tumor mass effect reduction and achieve symptom relief, as stated in a recent EANO consensus review [55].

Our survival analysis presents *several limitations*: its retrospective design always implies selection bias because patients in better clinical condition and with surgically amenable lesions are considered for second procedures. Furthermore, the relatively small sample size compared to other works made in a multicenter fashion [32] diminishes the power of the statistical analysis and reduces the capacity of assessing the role of reoperation in recurrent glioblastoma. However, our time-dependent evaluation will add data for potential future meta-analyses. Another advantage of a single institutional data source diminishes possible confounding effects between different neurosurgical units and allows for more similar groups to be contrasted. Most studies are retrospective because it is difficult to consider a prospective and randomized study assessing this question mainly due to ethical reasons. Quality of life is another concern taken into account by other colleagues [34] and is lacking in our retrospective analysis.

## Conclusion

Treatment for glioblastoma recurrence may be beyond the scope of  surgical means. To date, studies have provided conflicting results regarding second interventions in these cases. Moreover, the vast majority of the studies assessing this topic treat reoperation as a time-fixed covariate, which may overestimate survival data. A longer PFS with completion of the Stupp protocol seems to be the significant variable influencing OS, with little impact on PPS. Active treatment benefits survival after progression, while treatment choice is less relevant.

## Data Availability

The datasets used and/or analyzed during the current study are available from the corresponding author on reasonable request.

## Abbreviations

TMZ: Temozolomide
OS: Overall survival
PFS: Progression-free survival
PPS: Postprogression survival
BSC: Best supportive care
ECOG: Eastern Cooperative Oncology Group
IDH-1: Isocitrate dehydrogenase 1
MRI: Magnetic resonance imaging
SPSS: Statistical Package for the Social Sciences
5-ALA: 5-Aminolevulinic acid
RANO: Response assessment in neurooncology
MGMT: O^6^-Methylguanine-DNA-methyltransferase
EGFR: Endothelial growth factor receptor
HR: Hazard ratio
CI: Confidence interval
T2WI: T2-weighted image
EANO: European Society of Neuro-Oncology
